# Monitoring Depression Trends on Twitter During the COVID-19 Pandemic: Observational Study

**DOI:** 10.2196/26769

**Published:** 2021-07-18

**Authors:** Yipeng Zhang, Hanjia Lyu, Yubao Liu, Xiyang Zhang, Yu Wang, Jiebo Luo

**Affiliations:** 1 University of Rochester Rochester, NY United States; 2 University of Akron Akron, OH United States

**Keywords:** mental health, depression, social media, Twitter, data mining, natural language processing, transformers, COVID-19

## Abstract

**Background:**

The COVID-19 pandemic has affected people’s daily lives and has caused economic loss worldwide. Anecdotal evidence suggests that the pandemic has increased depression levels among the population. However, systematic studies of depression detection and monitoring during the pandemic are lacking.

**Objective:**

This study aims to develop a method to create a large-scale depression user data set in an automatic fashion so that the method is scalable and can be adapted to future events; verify the effectiveness of transformer-based deep learning language models in identifying depression users from their everyday language; examine psychological text features’ importance when used in depression classification; and, finally, use the model for monitoring the fluctuation of depression levels of different groups as the disease propagates.

**Methods:**

To study this subject, we designed an effective regular expression-based search method and created the largest English Twitter depression data set containing 2575 distinct identified users with depression and their past tweets. To examine the effect of depression on people’s Twitter language, we trained three transformer-based depression classification models on the data set, evaluated their performance with progressively increased training sizes, and compared the model’s tweet chunk-level and user-level performances. Furthermore, inspired by psychological studies, we created a fusion classifier that combines deep learning model scores with psychological text features and users’ demographic information, and investigated these features’ relations to depression signals. Finally, we demonstrated our model’s capability of monitoring both group-level and population-level depression trends by presenting two of its applications during the COVID-19 pandemic.

**Results:**

Our fusion model demonstrated an accuracy of 78.9% on a test set containing 446 people, half of which were identified as having depression. Conscientiousness, neuroticism, appearance of first person pronouns, talking about biological processes such as eat and sleep, talking about power, and exhibiting sadness were shown to be important features in depression classification. Further, when used for monitoring the depression trend, our model showed that depressive users, in general, responded to the pandemic later than the control group based on their tweets (n=500). It was also shown that three US states—New York, California, and Florida—shared a similar depression trend as the whole US population (n=9050). When compared to New York and California, people in Florida demonstrated a substantially lower level of depression.

**Conclusions:**

This study proposes an efficient method that can be used to analyze the depression level of different groups of people on Twitter. We hope this study can raise awareness among researchers and the public of COVID-19’s impact on people’s mental health. The noninvasive monitoring system can also be readily adapted to other big events besides COVID-19 and can be useful during future outbreaks.

## Introduction

### Background

COVID-19 is an infectious disease that has been spreading rapidly worldwide since early 2020. It was first identified on December 31, 2019, and was officially declared as a pandemic by the World Health Organization on March 11, 2020 [1]. As of September 15, 2020, COVID-19 has infected 216 countries, areas, or territories with over 29 million confirmed cases and 930,000 confirmed deaths [1]. In response to the pandemic, over 190 countries have issued nationwide closures of educational facilities [2], and many governments have issued flight restrictions and stay-at-home-orders, affecting the everyday lives of people worldwide.

Mental disorders were affecting approximately 380 million people of all ages worldwide before COVID-19 [3]. Previous psychological studies have shown that mental disorders lead to many negative outcomes including suicide [4,5]. However, these studies face two challenges. First, it is known that individuals with mental disorders are sometimes unwilling or ashamed to seek help [6]. Second, it is oftentimes infeasible for psychological studies to obtain and track a large sample of diagnosed individuals and perform statistically significant numerical analysis.

Multiple studies have investigated the economic and social impacts of COVID-19 [7,8], and various studies have shown that COVID-19 has greatly impacted people’s mental health worldwide. These studies found that there are higher rates of depression, anxiety, posttraumatic stress disorder (PTSD), and stress symptoms reported during COVID-19 than before [9]. Females, young age groups, students, and low education groups are especially susceptible to depression during the pandemic [9]. The pandemic negatively affected individuals’ mental health because of the changes that it brought to life. For example, it has been shown that after nationwide lockdowns people experienced high levels of stress because of social isolation [10]; the fact that a large proportion of the population is not wearing masks also makes people experience high levels of anxiety and depression [11]. For individuals with mental disorders, their need is amplified; the study by Hao et al [12] suggests that, during the pandemic, psychiatric patients reported more moderate to severe anger and impulsivity as well as concerns about their physical health, as opposed to the healthy controls, and that ideal remote mental health services such as telepsychiatry consultation and home delivery of medications could not be fully established due to the sudden lockdown [12].

Given this pressing situation, we would like to quantify mental health conditions of the general population during the pandemic. Nevertheless, the data source selection is critical for overcoming the two challenges mentioned previously. In the past decade, people have been increasingly relying on social media platforms such as Facebook, Twitter, and Instagram to express their feelings. Social media can thus serve as a resourceful medium for mining information about the public’s mental health conditions [13-17]. The public have long been known to search online for information about diseases and medical issues [18]. COVID-19 is no exception. Indeed, using social media, public opinions on personal face mask use [19] and COVID-19 vaccine uptake [20,21] have been investigated. Existing research has also studied the predictive power of online medical consultation, online medical appointment, and online medical search in forecasting regional outbreaks and found online medical consultation to be the most predicative [22]. Furthermore, a recent longitudinal study on the mental health of the Chinese population during the pandemic has found that dissemination of health information via radio was associated with higher levels of anxiety and depression, and suggested television and the internet as alternatives [23]. Therefore, we believe social media platforms like Twitter offer a solution to the challenges, as they enable us to perform a large-scale quantitative study on mental disorders in a noninvasive fashion.

**Figure 1:**
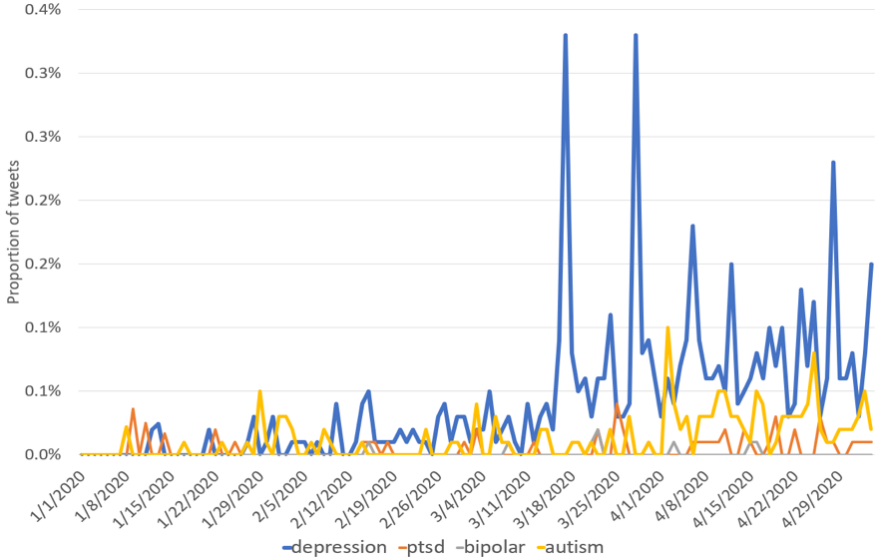
Density of Twitter coverage regarding “depression,” “ptsd,” “bipolar disorder,” and “autism.” ptsd: posttraumatic stress disorder.

As shown in Figure 1, we used data from the ForSight by Crimson Hexagonplot [24] to plot the word frequencies of several mental disorders on Twitter, including “depression,” “PTSD,” “bipolar disorder,” and “autism,” from January 1 to May 4, 2020. Note that we excluded false-positive tweets that contained misleading phrases such as “economic depression” or “great depression.” We noticed a rapid growth of the word frequencies of these mental disorders starting from March 17, when the pandemic spread across most of the world. Past research has suggested that depression is more pervasive than other psychological disorders during the COVID-19 period [9]. Similarly, we found that the word “depression” occurs substantially more frequently on Twitter compared to the other three mental disorders. Accordingly, depression is likely to be triggered most frequently by COVID-19, and we focused on understanding COVID-19’s impact on depression in this study.

### Prior Work

The potential of machine learning models for identifying Twitter users who have been diagnosed with depression was pioneered by De Choudhury et al [25], who analyzed how features obtained by Linguistic Inquiry and Word Count (LIWC) were related to depression signals on social media and how that can be used for user-level classification on a data set containing 171 depression users. The data was collected by designing surveys for volunteers through crowdsourcing. Following this work, Coppersmith et al [26] used LIWC, 1-gram language model, character 5-gram model, and user’s engagement on social media (user mention rate, tweet frequency, etc) to perform tweet-level classification on a data set containing 441 depression users.

The CLPsych 2015 Shared Task data set containing 447 diagnosed depression users [27] was published in 2015 and was favored by a wide range of studies [28-30]. The data was gathered by regular expression search in tweets in combination with manual annotation. Among these studies, the performance of traditional machine learning classification algorithms (decision trees, support vector machines [SVMs], naive Bayes, logistic regression) on 1-grams and 2-grams was investigated by Nadeem [30]; Jamil et al [28] used SVM on bag of words (BOW) and depression word count along with LIWC features and NRC sentiment features; Orabi et al [29] explored the performance of small deep neural network [architectures]—one-dimensional convolutional neural network (CNN) and bidirectional long short-term memory (BiLSTM) with context-aware attention—and achieved the best performance (87% accuracy) on the task.

The CLPsych 2019 Shared Task [31] focused on evaluating Reddit users’ suicide risk based on their posts, for which Matero et al [32] applied a pretrained Bidirectional Encoder Representations from Transformers (BERT) [33] embedding to encode the data. Suicide risk assessment on Spanish tweets was also studied by Ramírez-Cifuentes et al [34]. We argue that our task is different since few detected depressive Twitter users express suicide intent, while all the positive suicidal users in the suicide risk data sets should be viewed as in late stages of depression [35,36]. There are also some studies that performed depression detection on Reddit users [37-39] with sample sizes of less than 1300 Reddit posts. By contrast, we used the transformer-based models in our study, which have been shown to achieve state-of-the-art results in a wide range of natural language processing problems [33,40,41].

In addition to these two challenge data sets, several studies attempted to gather their own data of various forms. Tsugawa et al [42] performed analysis of models using BOW, latent Drichlet allocation (LDA) [43], and social media engagement features on a data set containing 81 Japanese-speaking depression Twitter users collected by crowdsourcing. Zhou et al [44] used ubiquitous multimodal sensors and performed in-depth analysis on users’ social media content, social network, webcam video, and user interaction on a sample of 5 depression users. Detecting depression from Spanish tweets using sentiment and emotion lexicons was used by Leis et al [45]. Zhang et al [46] performed observational analysis of the relationship between deteriorating depression and behavior changes when engaging with Google search and YouTube on 49 depressive college students. Shen et al [47] proposed a multimodal dictionary learning method that used topic, social media engagement, profile image, and emotional features to learn a latent feature dictionary that performed well on a data set of 1402 users with depression, the largest Twitter depression data set used to the best of our knowledge. Given the skyrocketing word density of “depression” in Figure 1, we show that a substantially larger depression data set can be quickly constructed from the COVID-19–related tweets within several months.

### Goal of the Study

Although the time series plots of keyword frequencies in Figure 1 offer an intuitive reading of depression’s general trend in the population, they are apparently filled with noise and lack plausible explanation to be an accurate representation. To generalize beyond keywords, we would like to train machine learning–based models to identify depression on social media. Reddit automatically gathers posts of the same topic into “subreddits”; however, as pointed out by Pirina and Çöltekin [38], labeling posts completely according to subreddit names causes categories to be topically specific and cannot be generalized to regular social media text. Moreover, depression prediction models can potentially be used on the population level [48], but none of the work mentioned in the previous section applied their models to the general Twitter population on the fly.

Therefore, the main objectives of this study are to develop a method to create a large-scale depression user data set in an automatic fashion so that the method is scalable and can be adapted to future events; to verify the effectiveness of transformer-based deep learning language models in identifying depression users from their everyday language; to further improve the depression classification model using explainable psychological text features and to examine their importance in classification; and, finally, to use the model for monitoring the fluctuation of depression levels of different groups as the disease propagates.

## Methods

### Data Collection

First, we identified users with depression from 41.3 million COVID-19–related tweets posted by about 36.6 million users from March 23 to April 18, 2020. We collected the COVID-19–related tweets using the keywords “corona,” “covid19,” “covid19,” “coronavirus,” “#Corona,” “#Covid_19,” and “#coronavirus.” From these tweets, we looked for signals that can tell whether the user has depression from both the text and the user profile description.

Empirically, we observed that many Twitter users with depression described themselves as “depression fighters” in their descriptions. Some of them may also post relevant tweets to declare that they have been diagnosed with depression. Inspired by Coppersmith et al [26], we used regular expressions to find these authors by examining their tweets and descriptions. Building upon their method, we further extended our regular expression search based on some patterns we noticed on manually identified depression users, in pursuit of efficacy. In tweets, we searched for phrases such as “I have/developed/got/suffer(ed) from X depression,” “my X depression,” “I’m healing from X depression,” and “I’m diagnosed with X depression,” where X is a descriptive word such as “severe” and “major” (X can be empty as well). In descriptions, we further added phrases such as “depression fighter/sufferer/survivor” to the regular expression list; we removed users that had “practitioner” and “counselor” in their descriptions to exclude mental health practitioners. The remaining users captured by the regular expressions were considered to have depression.

In the end, 2575 distinct Twitter users were classified into the depression group. Of 200 randomly sampled users in the depression set, 86% were labeled positive by human annotators. We randomly selected another 2575 distinct users so that depression-related terms did not appear in their past 200 tweets or descriptions as our control group. Users in this group were not considered to have depression (nondepression group). Once we found the targeted Twitter users, we used the Tweepy application programming interface (API) to retrieve the public tweets posted by these users within the last 3 months since the time of posting the depression-related tweet, with a maximum of 200 tweets per user. We chose 200 tweets because, on average, it is roughly the number of tweets posted by an individual within a 3-month time span, which is the length commonly adopted by previous work [25,26]. If a user was identified from the description, we limited the time scope from January 18 to April 18, 2020.

### Data Analysis

#### Personality

Previous psychological research has shown that the big five personality traits (openness, conscientiousness, extraversion, agreeableness, and neuroticism) are related to depression [49,50]. In particular, low extraversion, high neuroticism, and low conscientiousness were associated with depressive symptoms [50]. We estimated individuals’ personality scores using IBM’s Personality Insights service [51]. For each individual, we aggregated all their tweets into a single textual input and used the Personality Insights API to obtain the scores. The minimum number of words for using the API was 100, and we were able to retrieve 4697 (91.2%) of the 5150 users’ scores. Summary statistics are shown in Multimedia Appendix 1.

#### Sentiments

**Figure 2:**
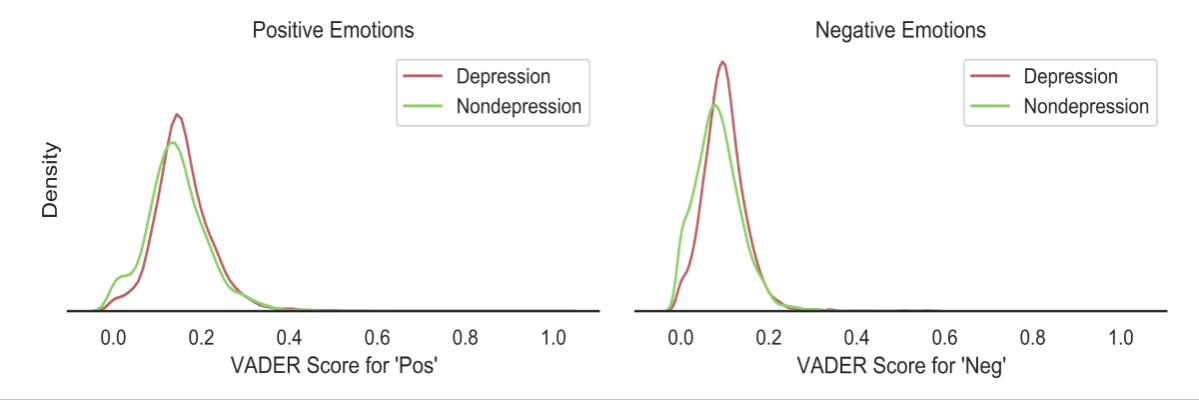
Distributions of positive and negative emotion scores among the depression and nondepression groups. VADER: Valence Aware Dictionary for Sentiment Reasoning.

Besides personality, we hypothesized that individuals’ sentiments and emotions could also reflect whether they were experiencing depression or not. Sentiment analysis is widely-used in deciphering people’s health and well-being from text data [52]. We estimated individuals’ sentiments using the Valence Aware Dictionary and Sentiment Reasoner (VADER). VADER is a lexicon and rule-based model developed by researchers from the Georgia Institute of Technology [53]. We aggregated a user’s tweets into a single chunk, applied VADER, and retrieved its scores for positive and negative emotions. In Figure 2, we reported the VADER score distributions of positive emotions and negative emotions among the depression and nondepression groups. Compared with individuals with no depression, those with depression tended to exhibit both stronger positive and negative emotions.

#### Demographics

Previous psychological studies have shown differences in depression rates among people of different ages and of different genders [54-56]. Research has shown a U-shaped relationship between age and depression, with depression reaching its lowest level around the age of 45 years [54]. Women are known to be substantially more likely to have depression [57]. To estimate the age and gender of the user, we adopted the M3-inference model proposed by Wang et al [58]. The M3 model performs multimodal analysis on a user’s profile image, username, and description. Following M3’s structure, we labeled each user with a binary gender label (as approximation) and a one-hot age label among four age intervals (≤18 years, 19-29 years, 30-39 years, ≥40 years), which were then used in our fusion model. Of the 5150 users, we were able to retrieve 5059 (98.2%) users’ demographic information.

#### Linguistic Inquiry Word Count

We used LIWC—a well-validated psycholinguistic dictionary [59]—to capture people’s psychological states by analyzing the contents of their tweets. LIWC is a dictionary-based linguistic analysis tool that can count the percentage of words that reflect different emotions, thinking styles, and social concerns, and captures people’s psychological states. Zhang et al [60] applied LIWC to the tweets of US working adults to analyze the influence of COVID-19 on their well-being; some LIWC features in college students’ YouTube and Google search logs have been shown to correlate with their Patient Health Questionnaire-9 depression scores [46]; Coppersmith et al [26] showed the relationship between the use of the first person pronoun (which is one of the LIWC features) and depression [26].

**Figure 3:**
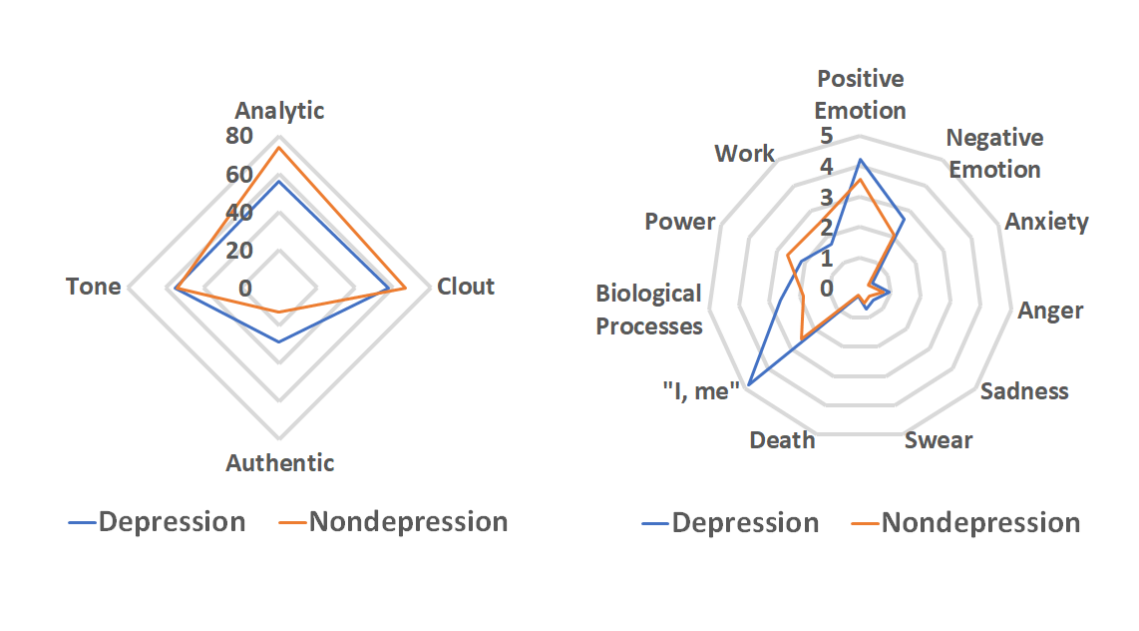
Linguistic profiles for the depression and nondepression tweets. LIWC: Linguistic Inquiry and Word Count.

We chose 8 features that were analyzed in previous works [26,61,62] and 7 other features that we found relevant to our study. Similar to the methods of Chen et al [63], we then applied LIWC to the concatenated tweets of individuals. Figure 3 shows the linguistic profiles for the tweets of the depression and nondepression groups. Both the depression and nondepression groups exhibited slightly positive tones, with negligible differences. The tweets of the nondepression group showed more analytical thinking, more clout, and less authentic expression than those of the depression group. The tweets of the depression group scored higher in both positive and negative emotion categories than the ones of the nondepression groups, which suggests a higher degree of immersion [64]. Moreover, the tweets of the depression group also showed more anxiety and anger emotions, and included more *swear* words—the *anxiety*, *anger*, and *swear* scores of the depression group were 50%, 22%, and 45% higher than that of the nondepression group, respectively—which is consistent with the findings of Coppersmith et al [26]. Death-related words appeared more frequently in the tweets of the depression group, which echoes Stirman and Pennebaker [62]. Similar to these 2 studies, we found more first person singular usage in the tweets of the depression group.

We also found that the tweets of the depression group expressed more sadness emotion and used words related to the biological process more frequently. Although there is no clear link between biological process–related words and depression, this finding shows that people with depression may pay more attention to their biological statuses. The *power* score for the tweets of the nondepression group was higher, which reflects a higher need for the power according to the findings of McClelland [65]. By comparing the *work* scores of the depression and nondepression groups, we found that the users of the nondepression group paid more attention to work-related issues as well.

#### Social Media Engagement

We used the proportion of tweets with mentions, number of responses, unique user mentions, user mentions, and tweets to measure the social media engagement of each user, as did Coppersmith et al [26]. To better understand the difference of social media engagement between the depression and nondepression groups, we added 0.1 to the number of responses, unique users mentions, users mentions, and tweets, and took the logarithm. By applying the Mann-Whitney rank test, we found that, except for the number of unique user mentions, other features were statistically different (*P*<.05) between the depression and nondepression groups. The users of the depression group posted more tweets and replied more. They tended to post fewer tweets with mentions, while the number of mentions for the depression group was larger, which suggests that when users of the depression group posted tweets to interact with other users, it involved more users.

### Modeling

#### Task Definition

We formulated our task as a classification task, where the model was trained to predict whether a particular tweet or a chunk of tweets comes from a user from the depression set. Note that not all tweets by people in the depression set were explicitly referring to depression per se. By definition, though, they were all posted by users with depression and were thus labeled true. To help improve the model’s generalizability, during training and testing, we excluded all the tweets used to identify the users with depression by regular expressions that contained trivial patterns and keywords. We assumed there were subtle differences in the language used between the depression and nondepression groups. Our goal was to build a model capable of capturing these subtleties and classifying users correctly.

#### Tweet Chunking and Preprocessing

We performed stratified random sampling on our data set. We first sampled 500 users to form our testing set. On the rest of the users, we progressively added users to the training sets and recorded the performance of the models trained on sets of 1000, 2000, and 4650 users. All the training and testing sets have a 1:1 (depression:nondepression) ratio.

Jamil et al [28] have shown that one single tweet does not contain enough signals to determine whether a user has depression. Thus, we concatenated consecutive tweets of the same user together to create tweet chunks of 250 words and labeled the chunks based on the user’s label. Given an input sentence, the transformer tokenizer first splits each word from the input sentence into *word-pieces* and then vectorizes them for computation. The 250 words roughly corresponded to the maximum 512 input word-pieces allowed by transformer-based language models including BERT [33] and Robustly Optimized BiLSTM Memory Pretraining Approach (RoBERTa) [40]. This limitation is due to the self-attention mechanism in the transformer, whose time complexity scales quadratically with the input sequence length.

We preprocessed the text using the tweet preprocessing pipeline proposed by Baziotis et al [66]. We adopted this method especially due to its capability of marking Twitter-specific text habits and converting them to special tokens such as “<allcaps>” (capitalized words), “<elongated>” (repeated letters), “<repeated>” (repeated words), etc. For example, “YESSSSS, I love it so much!!!” after preprocessing will be in the form of “Yes <allcaps> <elongated>, I love it so <repeated> much! <elongated>.”

After chunking and preprocessing, on average, each user had 6-7 text chunks, making the actual sizes of the 4650-user train-validation set and the 500-user testing set to be 29,315 and 3105, respectively. The preprocessed tweet chunk data sets were then passed to deep learning models for training.

#### Deep Learning Models

We used deep learning models to perform chunk-level classification. We set up two baseline models, multi-channel CNN and BiLSTM with context-aware attention (attention BiLSTM), as described in Orabi et al [29], which achieved the best performance on the CLPsych 2015 data set. We used the pretrained GloVe embedding (840B tokens, 300d vectors) [67] augmented with the special tokens added during preprocessing. The embedding weights were further trained jointly with the model. Recently, transformer-based deep learning language models have achieved state-of-the-art performance in multiple language modeling tasks. We trained three representative transformer-based sequence classification models—BERT [33], RoBERTa [40], and XLNet [41]—with their own pretrained tokenizers augmented with the special tokens for tokenization. We chose to use the base models for all of them since we found no noticeable performance gains using their larger counterparts.

#### Signal Fusion

We ran the models on all the tweet chunks of the same user and took the average of the confidence scores to get the user-level confidence score. There were 4163 (89.5%) out of 4650 users remaining in the training set and 446 (89.2%) out of 500 users in the testing set whose entire features were retrievable. We then passed different combinations of user-level scores (personality, VADER, demographics, engagement, LIWC, and average confidence) to machine learning classification algorithms including random forest, logistic regression, and SVM provided by the *scikit-learn* library [68]. We only used the explainable LIWC features mentioned in the data collection section for training the classifiers.

#### Training Details

During training, we randomly split the train-validation set to training and validation sets with a ratio of 9:1. We used Adam optimizer with a learning rate of 7e-3 and weight decay of 1e-4 for training attention BiLSTM. We used Adam optimizer with a learning rate of 5e-4 for training CNN. We used AdamW optimizer with a learning rate of 2e-5 for training BERT and RoBERTa, and 8e-6 for training XLNet. We used the cross-entropy loss for all our models during training. We used the stochastic gradient descent optimizer with adaptive learning rate, with initial learning rate as 0.1 for training SVM and logistic regression classifier. We recorded the models’ performances on the validation set after each epoch and kept the model with the highest accuracy and F1 scores while training until convergence. We manually selected the hyperparameters that gave the best accuracy and F1 scores on the deep learning models.

## Results

### Chunk-Level Classification

In Table 1, we report our classification results at the chunk level on the testing set. Our evaluation metrics included accuracy, F1 score, area under the receiver operating characteristic curve (AUC), precision, and recall. One immediate observation was that, regardless of the model type, the classification performance improved as we increased the size of our train-validation set. This shows that for building depression classification models it is imperative to have a large number of training samples. At the same time, it also confirms that the larger number of training samples in our experiments was indeed an advantage.

Another observation was the performance gain of transformer-based models over BiLSTM and CNN models. The CNN model slightly outperformed BiLSTM, which replicated the findings of Orabi et al [29]. We observed that BERT, RoBERTa, and XLnet invariably outperformed BiLSTM and CNN regardless of the size of our training set. In particular, the XLNet model recorded the best AUC and accuracy of all the models when trained with our full training set.

**Table 1:** Chunk-level performance (%) of all 5 models on the 500-user testing set using training-validation sets of different sizes.^a^

Model and training-validation set			Accuracy		F1		AUC^b^		Precision		Recall
**Attention BiLSTM^c^**											
	1000 users	70.7		69.0		76.5		70.9		67.3	
	2000 users	70.3		68.3		77.4		70.7		66.1	
	4650 users	72.7		71.6		79.3		72.1		71.1	
**CNN^d^**											
	1000 users	71.8		72.6		77.4		72.7		72.6	
	2000 users	72.8		74.5		80.3		72.2		76.9	
	4650 users	74.0		70.9		81.0		77.4		68.9	
**BERT^e^**											
	1000 users	72.7		74.4		79.8		72.0		76.9	
	2000 users	75.7		76.3		82.9		76.1		75.7	
	4650 users	76.5		77.5		83.9		76.3		78.8	
**RoBERTa^f^**											
	1000 users	74.4		75.7		82.0		74.2		77.3	
	2000 users	75.9		77.9		83.2		73.8		*82.5* ^g^	
	4650 users	76.2		*78.0*		84.1		74.4		81.9	
**XLNet**											
	1000 users	73.7		75.1		80.7		73.2		77.2	
	2000 users	74.6		76.8		82.6		72.6		81.5	
	4650 users	*77.1*		77.9		*84.4*		*77.5*		78.3	

^a^We used 0.5 as the threshold when calculating the scores.

^b^AUC: area under the receiver operating characteristic curve.

^c^BiLSTM: bidirectional long short-term memory.

^d^CNN: convolutional neural network.

^e^BERT: Bidirectional Encoder Representations from Transformers.

^f^RoBERTa: Robustly Optimized BiLSTM Pretraining Approach.

^g^Italics indicate the best performing model in each column.

### User-Level Classification

Next, we report our experiment results at the user level. Since XLNet trained on the 4650-user data set outperformed the other models, we took it for user-level performance comparison. Our experimental results demonstrated a substantial increase on the user-level scores of XLNet shown in Table 2 compared to the chunk-level score shown in Table 1. This indicates that more textual information of a user yields more reliable results on determining whether the user has depression. Building on the user-level XLNet scores, we further included VADER, demographic, engagement, personality, and LIWC scores as signals. We first used all features and compared the performance of random forest, logistic regression, and SVM. We noticed that SVM achieved the best scores on accuracy and F1, slightly surpassing logistic regression. Thus, we used SVM for testing the performance when using part of the features collected.

The results are shown in Table 2. The results have shown that using VADER, demographics, and social media engagement features alone does not help the classification by much. Classifiers using personality features and LIWC features perform relatively better. We then used these five feature groups and obtained a better result (accuracy 71.5%; F1 score 72.0%). However, the classifier was still outperformed by XLNet, showing that the transformer-based models indeed worked better on depressive Twitter text modeling compared with other approaches. We further increased the classifier’s performance by using all the features, namely, VADER, demographics, engagement, personality, and LIWC features, and the averaged XLNet confidence score; the performance of the three machine learning algorithms did not vary much, and the SVM classifier achieved the best accuracy (78.9%) and F1 (79.2%) scores.

**Figure 4:**
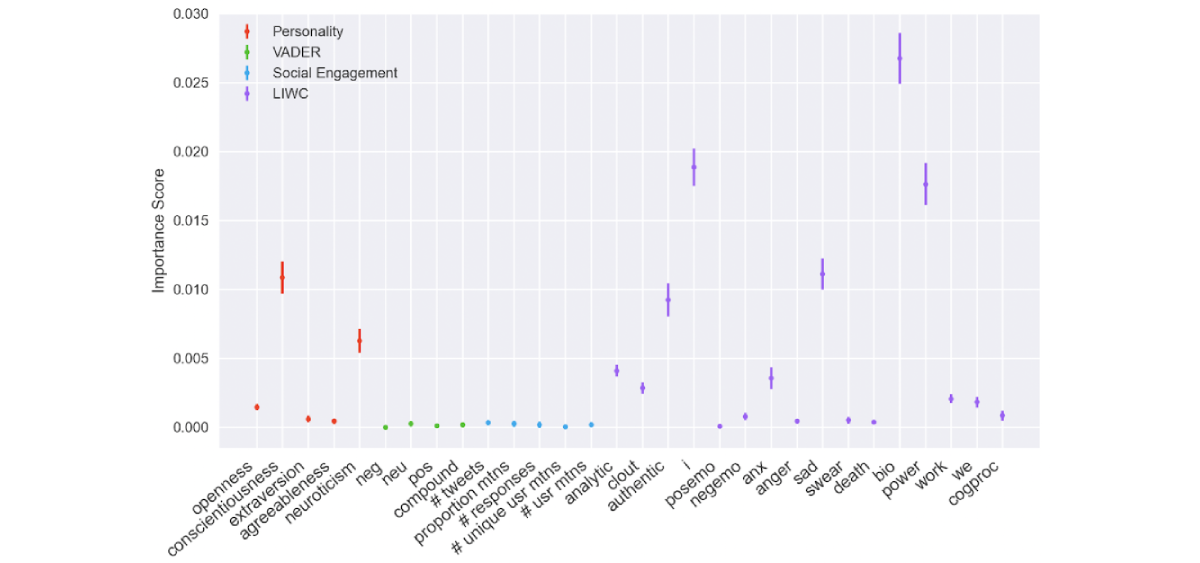
Permutation importance of different features. LIWC: Linguistic Inquiry and Word Count; VADER: Valence Aware Dictionary for Sentiment Reasoning.

In an attempt to investigate what specific textual features besides those extracted by XLNet have the most impact on depression classification, we calculated the permutation feature importance [69] on the trained random forest classifier using the VADER, engagement, personality, and LIWC features with 10 repeats. The importance scores of individual features are shown in Figure 4. Among the LIWC features, “i,” “bio,” “power,” “sad,” and “authentic” are shown to be important in classification. Among the five personality features, “conscientiousness” and “neuroticism” were shown to be closely related to depression cues. We did not observe a strong relation between VADER sentiment features or social media engagement features and the depression signals. As for the LIWC sentiment features, only “sad” and “anxiety” were shown to be relatively important. It is worth noting that LIWC’s “sad” and “anxiety” categories each referred to about 150 words. By contrast, more than 7500 words or features fell in to the negative category in VADER. The insignificance of VADER features can be attributed to the more focused nature of LIWC.

**Table 2:** User-level performance (%) using different features.

Features^a^	Accuracy	F1	AUC^b^
VADER^c^	54.9	61.7	54.6
Demographics	58.7	56.0	61.4
Engagement	58.7	62.3	61.7
Personality	64.8	67.8	72.4
LIWC^d^	70.6	70.8	76.0
V + D + E + P + L^e^	71.5	72.0	78.3
XLNet	78.1	77.9	84.9
All (random forest)	78.4	78.1	84.9
All (logistic regression)	78.3	78.5	*86.4* ^f^
All (SVM^g^)	*78.9*	*79.2*	86.1

^a^We used SVM for classifying individual features.

^b^AUC: area under the receiver operating characteristic curve.

^c^VADER: Valence Aware Dictionary and Sentiment Reasoner.

^d^LIWC: Linguistic Inquiry and Word Count.

^e^V + D + E + P + L: VADER + demographics + engagement + personality + LIWC.

^f^Italics indicate the best performing model in each column.

^g^SVM: support vector machine.

### Application Results

In this section, we report two COVID-19–related applications of our XLNet based depression classifier: (1) monitoring the evolution of depression levels among the depression group and the nondepression group, and (2) monitoring the depression level at the US country level and state level during the pandemic. We chose to use XLNet because of its simplicity as a stand-alone model, as it performed comparably to the fusion model.

#### Depression Monitoring on Depression and Nondepression Groups

**Figure 5:**
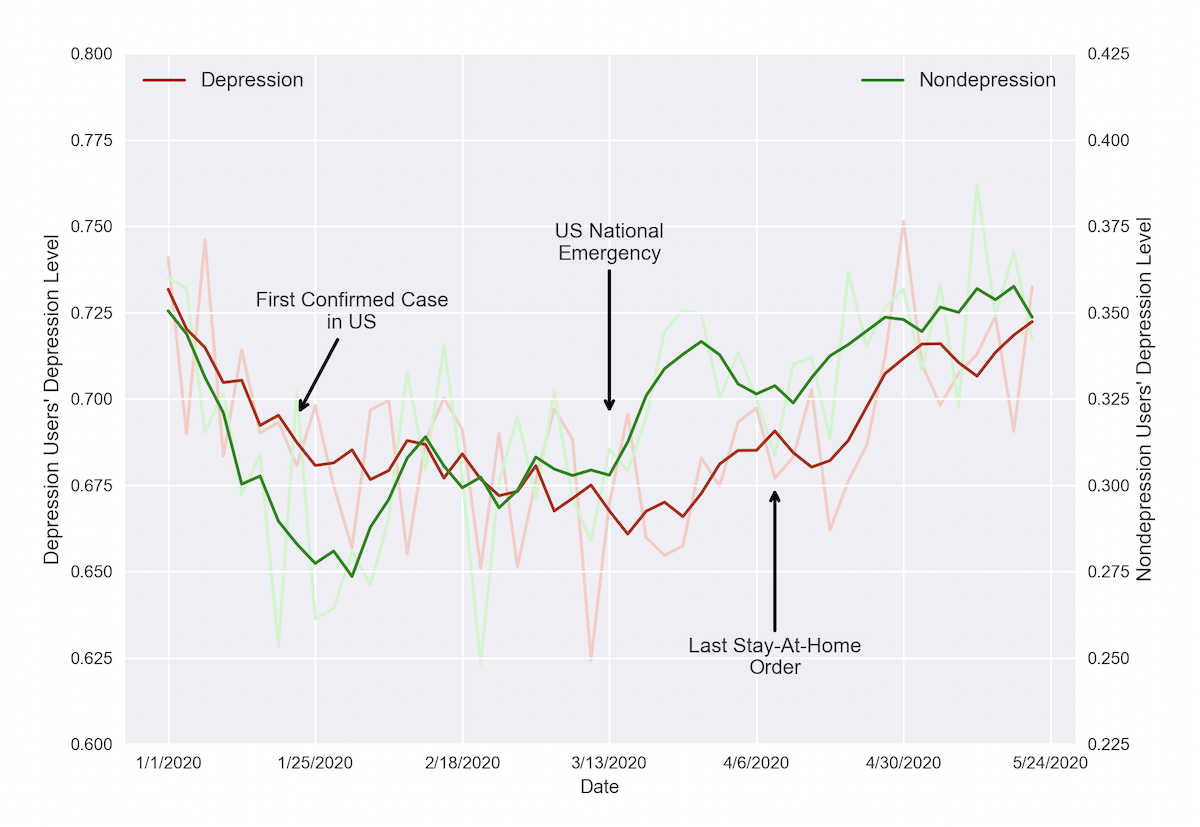
Aggregated depression level trends of the depression and nondepression groups from January 1 to May 22, 2020. Since users with depression have a substantially higher depression level, we used different y-axes for the 2 groups' depression levels to compare them side by side.

We took the 500 users from the testing set (n=500), along with their tweets from January 1 to May 22, 2020. We concatenated a user’s tweets consecutively from January 1 one by one until reaching 250 words and labeled this chunk’s date as the date of the author posting the tweet that was in the middle of the chunk. We grouped 3 days into a bin from January 1 and assigned the chunks to the bins according to the labeled date. We ran the XLNet model on the preprocessed tweet chunks and recorded the confidence scores. We trimmed the upper and lower 10% of the data to reduce the skew in the score distribution. We then took the mean of the scores for each time bin and plotted the depression trend shown in Figure 5. We further took a moving average of 5 time bins to smooth the curves.

Two immediate observations followed. First, depression level among users in the depression group was substantially higher than that in the nondepression group. This held across the entire observation period from early January to late May 2020. Second, and more importantly, the depression levels shared a strikingly similar trend among the two groups.

Delving deeper into these curves, we marked three important time points on the plot—the first confirmed case of COVID-19 in the United States (January 21, 2020), the US National Emergency announcement (March 13), and the last stay-at-home order issued (South Carolina, April 7). In January, both groups experienced a drop in depression scores. This may be caused by the fact that people’s mood usually hits its lowest in winter [70]. From the day when there was the first confirmed case in the United States to the day of the announcement of the US National Emergency, the trends of the depression and nondepression groups were different. The depression level of the depression group went down slightly, while the depression level of the nondepression group went up. Aided by psychological findings, we hypothesized that depressive users were less affected by negative events happening in the outside world because they focused on their own feelings and life events, since they were mostly affected by negative events that threatened them directly [71] and more interactions with the outside world gave them more negative feedback [72]. Moreover, the depression levels of the depression and nondepression groups both increased after the announcement of the US National Emergency.

**Figure 6:**
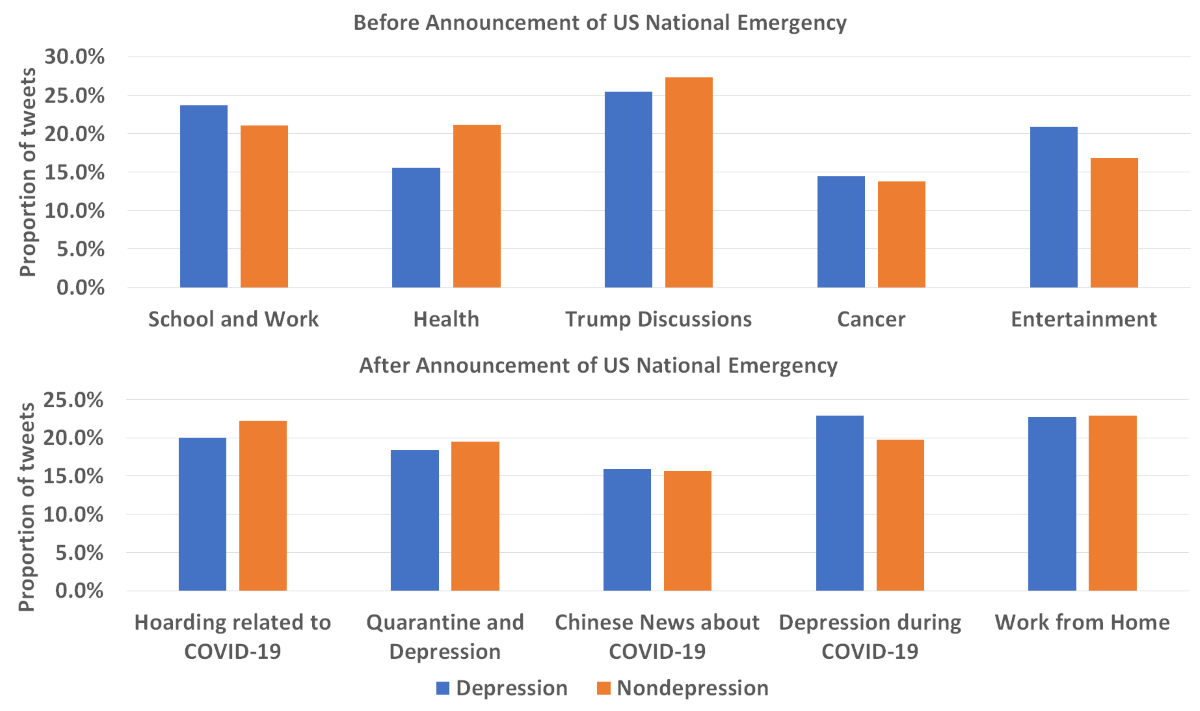
Topic distributions of depression and nondepression groups before and after the announcement of the US National Emergency.

To better understand the trend, we applied the LDA model to retrieve the topics before and after the announcement of the US National Emergency. Each chunk of the tweets was assigned 5 weights for each of the 5 topics. We labeled the topic of the highest weight as the dominant topic of this chunk of the tweets and counted the frequency of each topic shown in Figure 6. Details about the keywords of the topics are reported in Multimedia Appendix 1. Before the announcement, the two most frequent topics of the depression and nondepression groups were the discussions about US President Donald Trump and about school and work. The third most frequent topic of the nondepression group was about health while that of depression group was about entertainment. This supports the difference of the depression level trends of the two groups. After the announcement of the US National Emergency, the most frequent topic of the depression group was depression and anxiety during COVID-19, while this was the third most frequent topic of the nondepression group. Further, all 5 topics of each group were about COVID-19. This shows that, when people mostly talk about COVID-19, depression signals rise for both groups.

#### Aggregated Depression in COVID-19

**Figure 7:**
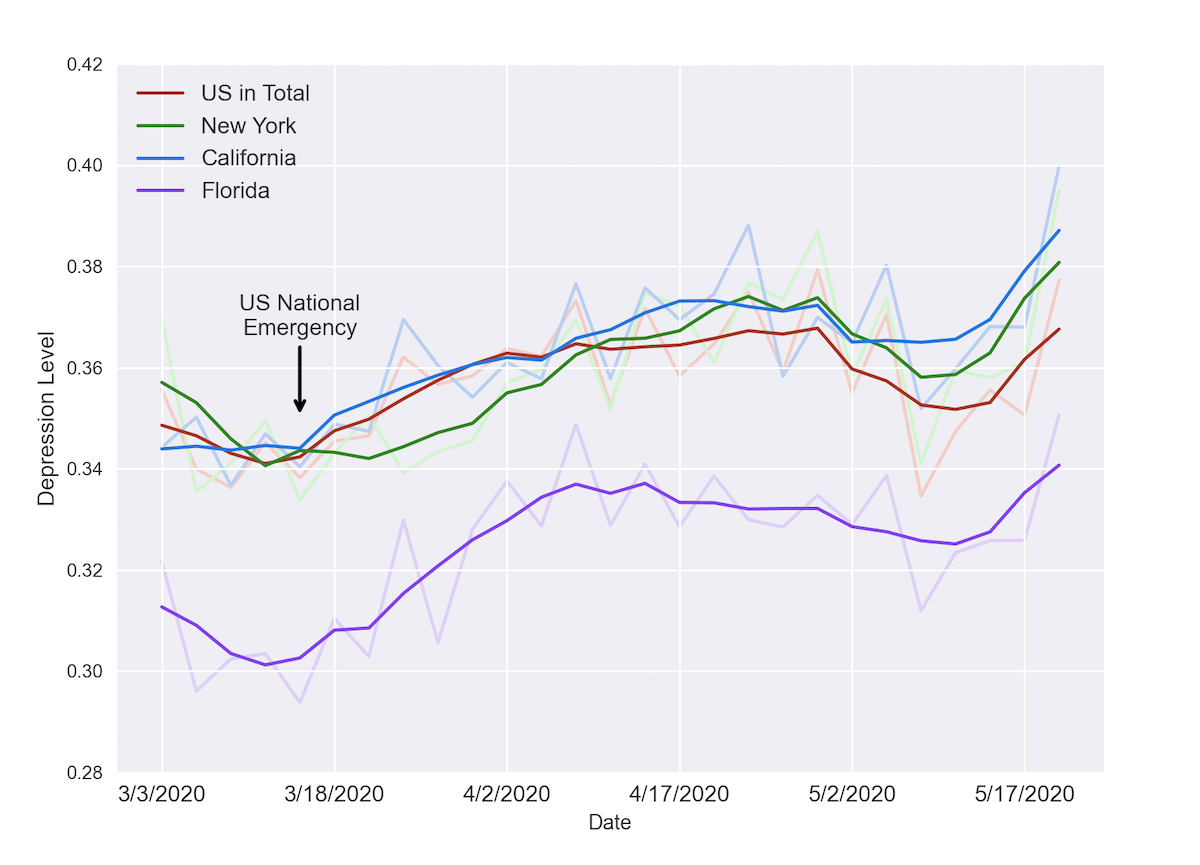
Aggregated depression level trends of the United States, New York, Califoria, and Florida after the announcement of the US National Emergency.

To investigate country-level and state-level depression trends during COVID-19, we randomly sampled users who had US state locations stated in their profiles and crawled their tweets between March 3 and May 22, 2020, the period right before and after the US announced a National Emergency on March 13. Using the same logic as in the previous section, we plotted the change of depression scores of 9050 geolocated users (n=9050) sampled from the 36.6 million users mentioned, excluding those used for training, as the country-level trend. For state-level comparison, we plotted the aggregated scores of three representative states—economical center New York on the East Coast that was highly affected by the virus, tech center California on the West Coast that was also struck hard by the virus, and the less affected tourism center Florida in the southeast. Each selected state had at least 550 users in the data set to validate our findings. Their depression levels are shown in Figure 7.

The first observation of the plot is that depression scores of all three states and the United States behaved similarly during the pandemic; they experienced a decrease right before the National Emergency; a steady increase after that; a slight decrease past April 23, 2020; and another sharp increase after May 10. We also noticed that the overall depression score of Florida was substantially lower than the US average and the other two states. Since Florida had a lower score both before and after the virus outbreak, we hypothesized that it has a lower depression level overall compared to the average US level irrespective of the pandemic.

**Figure 8:**
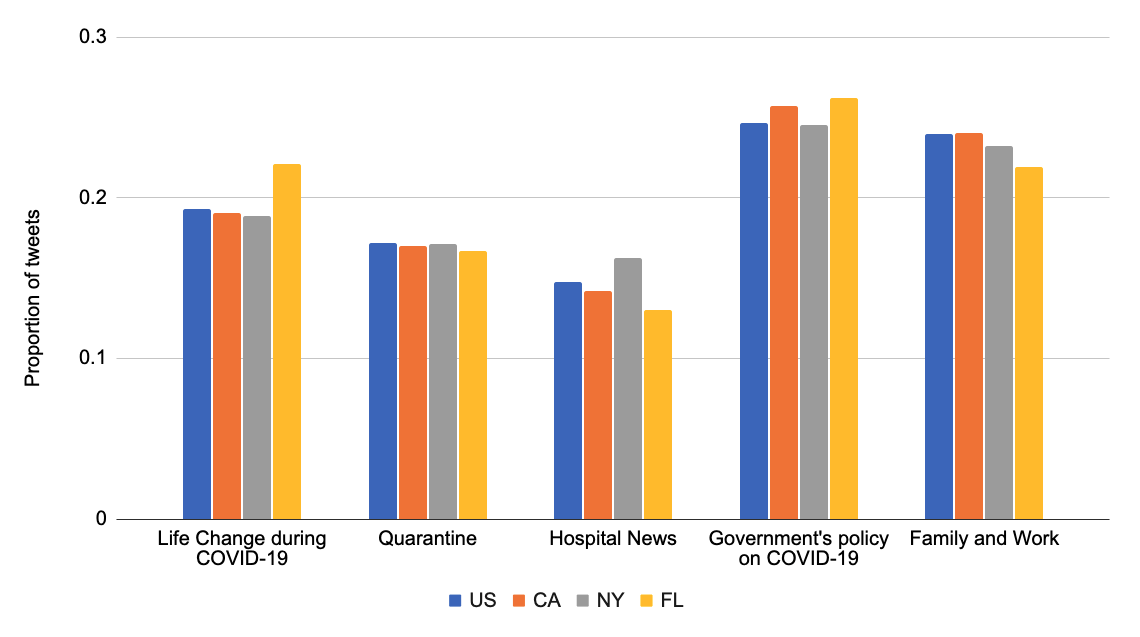
Distributions of the top 5 topics (state level) after the announcement of the US National Emergency.

We calculated the topics at the state level after the announcement of the US National Emergency. As shown in Figure 8, the most frequent topic was the government’s policy on COVID-19. California and Florida were the states that paid relatively more attention to this topic compared to the US average and New York State. Florida also talked more about the life change during COVID-19. Another finding was that people in New York talked more about the hospital news, likely because the state contained the majority of cases in the country by May 22, 2020 [73].

## Discussion

### Principal Results

In this study, we developed a practical pipeline that included first gathering and cleaning a large-scale Twitter depression classification data set quickly in response to an outbreak, then training an accurate depression signal detection model on this data set, and finally applying the model to monitoring public depression trends. We analyzed the depression level trends during the COVID-19 pandemic, which shed light on the psychological impacts of the pandemic. Our main results were fourfold and corresponded to the four objectives listed in the *Goal of the Study* section.

First, using a stringent yet effective regular expression-based search method, we constructed by far the largest data set with 5150 Twitter users, including half identified as depression users and half as control users, along with their tweets within the past 3 months and their Twitter activity data.

Second, we developed a chunking and regrouping method to construct 32,420 tweet chunks, with 250 words each in the data set. We progressively added data to our training set and showed experimentally that the performance of deep learning models improves as the size of the training set grows, which validates the importance of our data set size. We compared the models’ performances at the chunk level with the user level and observed further performance gain, which added credibility to our chunking method.

Third, we built a more accurate classification model (with 78.9% accuracy on n=449) upon the deep learning models along with linguistic analysis of dimensions including personality, LIWC, sentiment features, and demographic information. A permutation importance test showed that conscientiousness, neuroticism, appearance of first person pronouns, talking about biological processes such as eating and sleeping, talking about power, and exhibiting sadness are closely related to depression cues.

Finally, we showed the feasibility of the two proposed methods for monitoring the change of public depression levels as the disease propagates by aggregating individuals’ past tweets within a time frame. Our method can target different groups of people, and we showed the depression trends of identified depression and nondepression groups (n=500), and of groups at different geolocations (n=9050). The temporal trends showed that the nondepression group’s depression level rose earlier than that of the depression group, which we explained by psychological theories and LDA topics extracted from key time points. We also found that New York, California, Florida, and the United States in total all shared a similar depression trend, with Florida having a substantially lower depression level, which was also verified by LDA topic analysis.

### Practical Implications

Our study has practical implications. For example, upon detecting a rise in depression levels in a certain area, internet-based intervention services can be recommended by the social media platforms to the users. An intervention for depression commonly recommended is cognitive behavioral therapy (CBT), which is a type of therapy that targets one’s irrational thinking patterns and unadaptable behavioral patterns [74]. During the COVID-19 period, digital-based CBT can be adopted. It has shown to be effective in reducing symptoms of mental disorders [75,76]. At the same time, it is also cost-effective and practical during the pandemic [75]. In addition to digital-based CBT, social media–based suicide prevention messages have also shown to be effective [77] and can be sent to individuals at risk.

### Limitations

Although our data collection method is fast and fully automatic, we acknowledge that the same limitations exist as noted in detail by Coppersmith et al [26]. Specifically, the users with depression captured by us can only represent a subpopulation (those who use Twitter and are willing to disclose their conditions) of the general depression population, and we cannot guarantee that the control group was not contaminated.

### Comparison With Prior Work

The data set used in this study containing 2575 depression users was much larger than those used previously, which contained 1402 depression users at most. De Choudhury et al [48] demonstrated that depression prediction models can potentially be used at the population level. However, to the best of our knowledge, all Twitter user depression identification studies reviewed in the introduction section focus on either tweet-level or user-level classification rather than applying the model to analyzing the mental health trends of a large population. To our knowledge, we were also the first to apply the transformer-based models (BERT, RoBERTa, XLNet) to identifying depression users on Twitter using a large-scale data set and to monitor the public depression trend.

### Conclusions

COVID-19 has infected over 100 million people worldwide [1], virtually bringing the whole world to a halt. During this period, social media witnessed a spike in depression terms. Against this backdrop, we have developed transformer-based models trained with by far the largest data set on depression. We have analyzed our models’ performance in comparison to existing models and verified that the large training set we compiled was beneficial to improving the models’ performance. We further showed that our models can be readily applied to the monitoring of stress and depression trends of targeted groups over geographical entities such as states. We noticed substantial increases in depression signals as people talked more about COVID-19. We hope researchers and mental health practitioners find our models useful and that this study raises awareness of the mental health impacts of the pandemic.

## Appendix

Multimedia Appendix 1 Supplemental data statistics and tables.

## Abbreviations

API: application programming interface
AUC: area under the receiver operating characteristic curve
BERT: Bidirectional Encoder Representations from Transformers
BiLSTM: bidirectional long short-term memory
BOW: bag of words
CBT: cognitive behavioral therapy
CNN: convolutional neural network
LDA: latent Drichlet allocation
LIWC: Linguistic Inquiry and Word Count
PTSD: posttraumatic stress disorder
RoBERTa: Robustly Optimized Bidirectional Long Short-Term Memory Pretraining Approach
SVM: support vector machine
VADER: Valence Aware Dictionary and Sentiment Reasoner
