# Supplementary material for: Monitoring Depression Trends on Twitter During the COVID-19 Pandemic: Observational Study
Source: JMIR Infodemiology. 2021 Jul 18;1(1):e26769. doi: 10.2196/26769 (PMC8330892; doi:10.2196/26769)
Supplement: Multimedia Appendix 1 [file infodemiology_v1i1e26769_app1.docx]

# Supplemental Materials

## Supplemental Data Statistics

In Supplemental Table 1, we report the summary statistics of the personality estimates. We observe that all the estimates fall between 0 and 1. The standard deviations range from 0.24 to 0.28. Openness has the highest mean value at 0.61 and conscientiousness has the lowest mean value at 0.28.

**Supplemental Table 1.** Summary statistics of personality scores of users in the data set (N=4,697). SD stands for standard deviation.

|  | Min | Max | Mean | SD |
| --- | --- | --- | --- | --- |
| Openness | 0.00 | 1.00 | 0.61 | 0.28 |
| Conscientiousness | 0.00 | 1.00 | 0.28 | 0.26 |
| Extraversion | 0.00 | 1.00 | 0.32 | 0.24 |
| Agreeableness | 0.00 | 1.00 | 0.30 | 0.26 |
| Neuroticism | 0.00 | 1.00 | 0.56 | 0.28 |

In Supplemental Table 2, we further report the correlation coefficients between the personality variables. We observe that *extraversion* is highly correlated with *conscientiousness* and *agreeableness* (correlation coefficients > 0.45). Meanwhile, *neuroticism* is negatively correlated with *openness*, *conscientiousness*, and *extraversion*.

**Supplemental Table 2.** Correlation between the personality variables.

|  | Openness | Conscientiousness | Extraversion | Agreeableness | Neuroticism |
| --- | --- | --- | --- | --- | --- |
| Openness | 1 |  |  |  |  |
| Conscientiousness | 0.238^b^ | 1 |  |  |  |
| Extraversion | 0.265^b^ | 0.475^b^ | 1 |  |  |
| Agreeableness | -0.0768^b^ | 0.441^b^ | 0.469^b^ | 1 |  |
| Neuroticism | -0.232^b^ | -0.279^b^ | -0.285^b^ | 0.0460^a^ | 1 |
| ^a^*P* < .01, ^b^*P* < .001 | | | | | |

## LDA Topics

We use the LDA model provided by the *gensim* python library to model the topics of the tweets. To better understand the topics, we remove all the adjectives, adverbs and verbs from the text, and only keep the nouns. The number of the topics is set to be 5. In total we have trained three LDA models: one for tweets of DP/NP groups before the announcement of U.S. National Emergency (Model 1, N=500), one for tweets of DP/NP groups after the announcement of U.S. National Emergency (Model 2, N=500), and one for national and state level tweets of geo-located users between March 3rd, 2020 and May 22nd, 2020 (Model 3, N>550). Supplemental Table 3 shows the top 15 keywords for each topic.

**Supplemental Table 3.** Topics generated by the LDA model HG tweets.

| **Model** | **Topics** | **Top 15 Topic Words** |
| --- | --- | --- |
| **1** | School and Work | time, day, people, anyone, thing, support, work, man, class, today, school, week, watch, love, dog |
|  | Health | day, world, time, week, thank, today, ko, health, hope, video, news, something, hell, job, people |
|  | Trump Discussions | people, life, time, day, work, love, trump, lot, man, today, person, someone, thing, way, hope |
|  | Cancer | time, year, people, day, work, way, tweet, city, cancer, head, friend, death, today, love, problem |
|  | Entertainment | love, people, time, way, song, something, day, man nothing, everyone, tonight, year, today, game, guy |
| **2** | Hoarding Related to COVID-19 | time, dog, people, man, everything, call, work, way, covid, hope, news, food, night, thank, someone |
|  | Quarantine and Depression | time, work, love, day, people, something, man, thank, thing, hope, everyone, life, quarantine, house, home |
|  | Chinese News about COVID-19 | china, people, time, love, day, street, song, name, person, trump, news, government, expert, virus, dey |
|  | Depression during COVID-19 | people, today, time, day, covid, work, home, love, virus, man, hope, tweet, thing, depression, everyone |
|  | Work from Home | day, people, time, love, everyone, job, quarantine, today, year, thing, life, home, way, week, video |
| **3** | Life Change during COVID-19 | day, today, march, morning, week, life, run, friend, show, death, man, place, mayor, order, change |
|  | Quarantine | day, time, love, quarantine, today, ass, night, year, thank, tomorrow, house, video, game, life, miss |
|  | Hospital News | covid, state, home, work, health, news, county, week, hospital, testing, today, day, world, time, order |
|  | Government's Policy on COVID-19 | trump, people, president, time, virus, care, vote, country, china, money, need, medium, nothing, job, everyone |
|  | Family and Work | time, people, love, man, way, job, thank, day, work, lot, someone, something, thing, family, today |
